# Emerging Infectious Diseases, Antimicrobial Resistance and Millennium Development Goals: Resolving the Challenges through One Health

**DOI:** 10.5195/cajgh.2013.76

**Published:** 2013-10-01

**Authors:** G. V. Asokan, R. K. Kasimanickam

**Affiliations:** 1 Public Health Program, College of Health Sciences, University of Bahrain, Manama, Bahrain; 2 Department of Veterinary Clinical Sciences, College of Veterinary Medicine, Washington State University, Pullman, WA

**Keywords:** Emerging infectious diseases, Zoonoses, Antimicrobial resistance, Millennium development goals, One health

## Abstract

Most emerging infectious diseases are zoonoses, which could severely hamper reaching the targets of millennium development goals (MDG). Five out of the total eight MDG’s are strongly associated with the Emerging Infectious Diseases (EIDs). Recent emergence and dissemination of drug-resistant pathogens has accelerated and prevent reaching the targets of MDG, with shrinking of therapeutic arsenal, mostly due to antimicrobial resistance (AMR). World Health Organization (WHO has identified AMR as 1 of the 3 greatest threats to global health.

Until now, methicillin-resistant staphylococcus aureus (MRSA) and vancomycin-resistant enterococci (VRE) have been observed in hospital-acquired infections. In India, within a span of three years, New Delhi metallo-β-lactamase prevalence has risen from three percent in hospitals to twenty- fifty percent and is found to be colistin resistant as well. Routine use of antimicrobials in animal husbandry accounts for more than 50% in tonnage of all antimicrobial production to promote growth and prophylaxis. This has consequences to human health and environmental contamination with a profound impact on the environmental microbiome, resulting in resistance.

Antibiotic development is now considered a global health crisis. The average time required to receive regulatory approval is 7.2 years. Moreover, the clinical approval success is only 16%. To overcome resistance in antimicrobials, intersectoral partnerships among medical, veterinary, and environmental disciplines, with specific epidemiological, diagnostic, and therapeutic approaches are needed. Joint efforts under “One Health”, beyond individual professional boundaries are required to stop antimicrobial resistance against zoonoses (EID) and reach the MDG.

Emerging infectious diseases (EID), mostly zoonoses, pose enormous threats, which could severely hamper reaching the targets of the health-related United Nations Millennium Development Goals (MDG) set for 2015. 1 The following five out of the total eight MDG’s are strongly associated with the EIDs:

1. Reducing child mortality rates;
2. Improving maternal health;
3. Combating HIV/AIDS, malaria, and other diseases;
4. Ensuring environmental sustainability; and
5. Developing a global partnership for development.

Of all known infectious diseases, zoonoses constitute about 60%. Within emerging infectious diseases, approximately 75% are of zoonotic origin. By classification, 40% of fungi, 50% of bacteria, 70% of protozoa, 80% of viruses, and 95% of helminths that infect human beings are zoonotic. It has been recognized that more than 50% of human pathogens can infect other vertebrate hosts. 2 Only 100 of the approximately 400 known emerging pathogens occur as human pathogens. 3 Among the marine mammal pathogens at least 49% are zoonotic, and 28% are emerging zoonoses. 4

Emerging zoonoses, such as hendra, nipha, avian influenza, and severe acquired respiratory syndrome (SARS) are a growing threat to global health and have caused huge economic loss in the past 20 years. There is poor understanding of how zoonotic pathogens evolve from natural ecology and cause disease, and how various circumstances, such as animal production, extraction of natural resources, and antimicrobial application alter the dynamics of disease exposure to human beings. 5 To counter the shared risks between animals and humans, the concept of “one health” was developed during the early 21^st^ century and is based on a systems approach. 6 One health has been defined as “the collaborative effort of multiple disciplines working locally, nationally, and globally to attain optimal health for people, animals, and the environment.” 7 The purpose of this review is to look at the challenges faced due to antimicrobial resistance in EID particularly zoonoses and find solutions under a “one health” approach to reach the targets of the MDG.

## Antimicrobial resistance

Microorganisms have evolved over the ages and have an innate ability to survive by developing resistance to antimicrobial compounds administered. Antimicrobial resistance (AMR), sometimes known as drug resistance, occurs when microorganisms such as bacteria, viruses, fungi and parasites change ways to render the existing standard medications such as antibacterials (antibiotics -MRSA), antifungals (candida resistance to fluconazole), antivirals (H1N1 resistance to oseltamivir), and antiparasitics (Chloroquine resistance to malarial parasite) ineffective.

In recent times, the emergence and dissemination of drug-resistant pathogens has accelerated, proving to be global, extremely dangerous, and preventing reach of the set targets of MDG. With no borders between ecosystems, the spread of drug resistance is linked to the following: human life activity and travel, animals and the food trade, wild animals, migration, transportation, as well as water and wind flow. Further, EID are becoming untreatable and uncontrollable due to shrinking of the existing therapeutic arsenal, mostly due to AMR. Sensing the public health threat and having identified AMR as one of the three greatest threats to global health, the World Health Organization (WHO) announced the theme for World Health Day 2011 as “Antimicrobial resistance: no action today, no cure tomorrow.” 8 Moreover, the emergence of “superbugs” occurs when microorganisms become resistant to most antimicrobials currently available. These resistant superbug infections are of great concern, since this may spread and cause death, placing burden on health care expenditures.

### AMR in humans

In humans, antimicrobials are commonly used for the treatment and control of infectious diseases that address three of the eight MDG, namely: reducing child mortality rates, improving maternal health and combating HIV/AIDS, malaria, and other diseases. Besides, antimicrobials are used for treatment and prophylaxis of complex surgeries, intensive care, organ transplants, care of premature babies and the elderly, and survival of the immunosuppressed.

Severe problems of AMR are associated with multidrug-resistant tuberculosis and extensively drug-resistant tuberculosis. Rarely, mycobacterial infections of livestock are treated with antimicrobials, which could therefore add to the pool of tuberculosis. Falciparum malaria parasites resistant to artemisinins are emerging. New resistance mechanisms, such as the beta-lactamase (New Delhi metallo-β-lactamase- NDM-1) have emerged among several gram-negative bacilli. The beta-lactamase resistant strains of *Escherichia coli* from India have spread to other countries. 9

Until recently, such completely resistant bacteria have only been found in hospitals, such as methicillin-resistant staphylococcus aureus (MRSA) and vancomycin-resistant enterococci (VRE) in hospital-acquired infections. In India, within a span of three years, NDM-1 prevalence has gone up from three percent in hospitals to 20 to 50 percent, and patients were found to be resistant to colistin which is used against multi resistant gram negative bacteria. 10 Other causes of AMR in humans include: over-the-counter selling of antimicrobials without a licensed physicians prescriptions has been rampant mostly in the developing world and pharmaceutical incentives to the physicians for prescriptions.

### AMR in animals and environment

Manifestation of antimicrobial resistance in veterinary medicine is intricate because of the number of animal species, the diversity of environmental conditions in which the animals are reared, the differences in the microbes involved and pathogenicity mechanisms, and the complex epidemiology. 11 In most parts of the world, routine use of antimicrobials in animal husbandry account for more than 50% of the tonnage of all antimicrobial production to promote growth and for prophylaxis in food-producing animals of cattle, poultry, swine, fish, and honeybee. In the United States alone, 80% of all antibiotics sold are administered to food producing animals for growth promotion and prophylaxis. 12 It has been estimated that antibiotic use in animals and fish is far greater than the usage in humans (as much as 1,000-fold higher). 13 Risk quantification by the use of antimicrobials in animal husbandry is not possible due to the vast dispersal area from run-off and other sources of environmental contamination. 14

Further, use of antimicrobial agents in food-producing animals has also contributed to the development of resistant pathogens with resistance genes. The direct effect was observed more than 35 years ago, with high rates of AMR in the intestinal flora of farm animals and farmers. 15 Molecular detection tools have shown that resistant bacteria in food producing animals reach consumers through meat products. 16 Usually, resistant microbes in animals are transferred to people, not only through consumption of food but also through direct contact with food-producing animals or through environmental spread. As an indirect impact, it has been observed that up to 90% of antimicrobials given to animals are excreted in urine and stool and then widely dispersed through fertilizer, surface runoff, and groundwater, with a profound impact on the environmental microbiome. This has implications on two MDG’s namely: ensuring environmental sustainability, and developing a global partnership for development.

Various environmental samples of different geological age have proved that resistance genes in the environment are higher than those found in pathogens, and have existed for thousands of years. The word ‘resistome’ refers to the population of resistant genes in nature. 16,17 This has resulted in human infections with resistant microbes to antimicrobial agents and is difficult or impossible to cure. Extensive use of invasive procedures, and high rates of antimicrobial use, results in a nosocomial “environmental resistome.” Of special concern is resistance to antimicrobial agents classified by the World Health Organization (WHO) as critically important for human medicine, such as fluoroquinolones, third- and fourth-generation cephalosporins, and macrolides. 18

### Research and development of new antimicrobial agents

The discovery of antibiotics in the 1930s and 1940s transformed medicine from a diagnostic to a therapeutic discipline. 19 Sulphonamides and penicillin came into use in the 1940s. Selman Waksman discovered streptomycin in 1943 which propelled important findings related to the ‘secondary metabolites’ produced by actinomycetes, 20 and the next 40 years was considered as the golden era of antimicrobials. New classes of antibiotics were discovered, existing antibiotics were modified, and synthetic components were constantly tailored to combat emerging AMR by improving the clinical qualities. Until the late 1980s, the problem was not considered significant as many new substances were developed and marketed when resistance rendered existing drugs inefficient.

However, views changed, as few new antibiotics have been introduced since the 1990s. In addition, financial challenges for antibiotic development showed a poor investment return, as they are taken as a short course to cure the targeted disease. In contrast, drugs that treat non communicable diseases, such as high blood pressure, are continuously taken for the patient’s life. This can be illustrated in terms of net present value (NPV): at discovery, an antibiotic has a NPV of –$50 million and are generally priced at a peak charge of $1,000–$3,000 per course, whereas, NPV for a new musculoskeletal drug is estimated to a +$1 billion and a chemotherapy for cancer sometimes costs >$80, 000. 21

Antibiotic development is now considered a global health crisis. The average time required to take a product from the start of clinical testing to regulatory approval is 7.2 years, this excludes phases of discovery, research, preclinical and animal testing. 22 Moreover, the clinical approval success rate (the likelihood that a compound entering clinical testing will eventually reach the marketplace) is only 16%. 23

A report published in 2004 showed that, of 506 drugs in development by 15 large pharmaceutical companies and seven major biotechnology companies, only six were antibiotics. This has declined further; by 2008, eight of the 15 major pharmaceutical companies had abandoned antibiotic discovery programs and two others had reduced them. 24 Approval of new antibacterial agents by the United States Food and Drug administration has shown a decrease of 56% between 1998 and 2002, and a 75% decrease in systemic antibacterials approved from 1983 through 2007; 25 evidence of continued decrease in approvals was noticed between 2003 and 2007. 26 Yet another discouraging report mentions that no new class of antibiotics for gram negative bacilli has been found in the last four decades; only 2 drugs with new microbial targets (linezolid and daptomycin) have been introduced since 1998. 27 Recently, a study on antibiotic development involving small firms as well as large pharmaceutical companies revealed that only 15 of 167 antibiotics under development had a new mechanism of action. 28 Subsequently, a review identified that eight out of nine synthetic compounds are derived from quinolones, a class of antibiotic that may only require minor chromosomal mutations to gain resistance. 29 It is estimated that over the next 5–10 years, the number of approved antibacterials will plateau at a level similar to that of the past 5 years which may approximate 1 drug per year. 30

**Figure 1:**
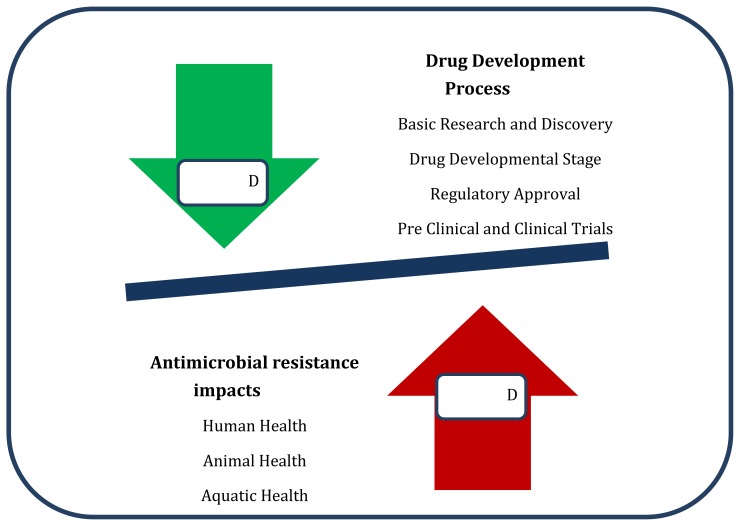
Association between disease status and antimicrobials.

The association between disease status and antimicrobials is illustrated in Figure 1. While the drug development process diminishes the disease load in a population it is countered by an increased disease load in humans, animals including aquatic and the environment by AMR.

## Conclusion

Most of the emerging infectious diseases are zoonoses, which have a direct influence on the majority of MDG. The emergence and spreading of drug-resistant pathogens, particularly zoonoses, has accelerated due to overuse, not following the prescribed length of use, misuse, and abuse of antimicrobials. More essential medicines are failing. The speed with which these drugs fail and are being lost far outpaces the development of replacement drugs. AMR challenges control of infectious diseases, hampers MDG, threatens a return to the pre-antibiotic era, increases the health care budget, jeopardizes health-care gains achieved, compromises health security, and damages trade and economies.

In order to reach the MDG, antimicrobial conservation must be one of the essential strategies. With limited health care resources, achieving a balance between conserving the effectiveness of existing antibacterial drugs and developing new ones is attracting attention among policy circles that will best serve public health, such as in viral respiratory tract infections a “delayed prescription” (any prescription for an antimicrobial where the patient is advised to delay getting the antimicrobial agent). Preventing use of these drugs in self-resolving disease with uncertain evidence of effectiveness would limit the spread of antimicrobial resistance. In addition, maximizing hospital infection-control practices, antimicrobial surveillance and restricting the use of antibiotics in humans and animal husbandry can achieve conservation of antimicrobials.

Finally, to overcome microbial evolution, issues in antibiotic pipeline, and resistance in antimicrobials, intersectoral partnerships in research among medical, veterinary medical, and environmental disciplines, with specific epidemiological, diagnostic and therapeutic approaches needed. The “10 × ‘20” initiative of the Infectious Disease Society of America is one such initiative. Diagnostic tests to identify resistant organisms with new approaches (i.e. procalcitonin levels) are markers to facilitate decisions for when to use or stop antibiotics as these levels reflect bacterial replication. Evidence has come from a meta-analysis and has shown that decisions guided by procalcitonin levels reduced antibiotic use by 51% without altering outcome. 31 Joint efforts are required under the “One Health” multidisciplinary/interdisciplinary collaborative approach, beyond individual professional boundaries, to stop antimicrobial resistance against zoonoses (EID) and reach the MDG.
